# From Entrepreneurship Education, Government Support, and Global Competence to Entrepreneurial Behavior: The Serial Double Mediating Effect of the Self-Efficacy and Entrepreneurial Intention

**DOI:** 10.3389/fpsyg.2022.838232

**Published:** 2022-04-12

**Authors:** Jinzi Zhang, Bing Li, Yanning Zhang, Chi Gong, Ziyang Liu

**Affiliations:** ^1^Department of Global Business, Kyonggi University, Suwon, South Korea; ^2^College of Art and Design, Shenzhen University, Shenzhen, China

**Keywords:** entrepreneurship education, global competence, government support, entrepreneurial self-efficacy, entrepreneurial intention, entrepreneurial behavior

## Abstract

Entrepreneurship plays a significant role in promoting the social and economic development of a country. At present, entrepreneurship education is widely carried out in universities and colleges in order to improve students’ entrepreneurial ability, and then to provide support for the formation of a comprehensive entrepreneurial situation. As entrepreneurship education has gradually become a hot topic of teaching for innovation and entrepreneurship education of international students, studies on the influencing mechanism of entrepreneurship education of international students in relation to their entrepreneurial behavior are conducive to providing theoretical basis and empirical evidence for international students’ entrepreneurship education, so as to pertinently guide international students’ innovation and entrepreneurship practice. This study aims to explore the influence of entrepreneurship education, government support for entrepreneurship, global competence, entrepreneurial self-efficacy, and entrepreneurial intention on the entrepreneurial behavior of international students in the Republic of Korea (“Korea”). It summarized and drew on the results of the existing literature research. According to the contents and points of research, this study takes the international students studying in Korea as the sample and uses statistical analysis software, SPSS 22.0 and AMOS 24.0, to establish a structural equation model to conduct empirical study on the influencing mechanism of entrepreneurial behavior of international students in Korea, so as to better understand the influence of entrepreneurship education in Korean universities and colleges on entrepreneurial behavior of international students in Korea. Based on the analysis results, this study puts forward the theoretical basis for the policies related to effective management of entrepreneurship, which will help alleviate the unemployment of young people studying in Korea and the tight labor market supply and demand.

## Introduction

As prolonged economic recession, young people’s unemployment, early retirement, aging population, COVID-19 pandemic and other social problems are intertwined, and the re-employment and retirement age are not guaranteed, the interest in and the necessity of entrepreneurship are growing day by day. Among the above-mentioned, young people’s unemployment is a problem that most countries of Organization for Economic Co-operation and Development (OECD) are also facing, and is a widespread social phenomenon in capitalist countries (Kim and Geum, 2020). With the increase in automation and outsourcing services, direct employment has decreased, and the pay gap between large companies and SMEs, and between regular employees and informal employees leads young people to deliberately avoid working in SMEs or as an informal employee, which has become a main cause for the increase of young people’s unemployment rate. Young people’s unemployment has not only become a cause of social unrest, but has also given rise to avoidance of marriage and low birthrate, which has become a social problem. Therefore, promoting entrepreneurship, as one of the measures to address young people’s unemployment (Shefiu, 2016), is also helpful to create employment opportunities, improve national competitiveness, and promote economic development.

At present, the Korean government is actively supporting the work of creating more jobs for the young people who are caught in employment difficulties in the face of structural shortage of labor. According to the information released by the Small and Medium Enterprise Agency, a large amount of money has been invested in entrepreneurship projects supported by the government in 2020, specifically, 6.668 billion won in the youth Bizcool (Entrepreneur) project, 4.46 billion won in technical entrepreneurship education to the seniors, and 1.51 trillion won in general entrepreneurship funds. Government support for entrepreneurship has become an important policy direction to address the structural problems of job shortage and young people’s unemployment (Lee and Yi, 2019). Despite such large-scale government support, effective entrepreneurship education, and entrepreneurship performance are still insufficient. Entrepreneurial enterprises still lack such kind of practical entrepreneurship education that can effectively utilize the resources provided by the government and achieve good results.

In recent years, studying abroad has become a core experience of adolescents and the international market has paid more attention to the mobility of international students. In fact, a steadily increasing number of students are interested in completing part of their academic education abroad (Amendola and Restaino, 2017). Studying abroad is not only a way for students from different countries to flow and exchange, but also an opportunity to cultivate talents with global competence for the country and the world. Students who study abroad can obtain quality education and master skills that cannot be taught by families, and are easier to access to local or global labor markets. At the same time, it is also considered to be one of the ways to improve the employment possibility in an increasingly globalized labor market (Um and Zhang, 2021). Therefore, it is necessary to conduct entrepreneurship-related research on international students who are more likely to conduct entrepreneurial activities in the future.

Many previous studies have investigated the relationship between entrepreneurs’ (international students’) personal characteristics (internal factors) and environmental characteristics (external factors) in the formation of entrepreneurial behavior, such as risk sensitivity (Kim and Kim, 2021), need for achievement (Mat et al., 2015), self-efficacy (Naktiyok et al., 2010), and entrepreneurial support (Jung, 2018). Kim and Lee (2020) pointed out that entrepreneurship education in universities and colleges and government support, as external factors, have a significant positive influence on entrepreneurial intention. Many scholars believe that environmental characteristics are important when starting a business, but entrepreneurs’ personal characteristics are no less important than, and sometimes even more important than environmental characteristics (Crant, 1996; Lee and Lee, 2016). Some researchers found that global competence among international students’ internal factors has a significant positive influence on entrepreneurial intention (Lee and Lee, 2016; Um and Zhang, 2021). Nevertheless, there is little and limited literature on the influence of international students’ global competence on entrepreneurial behavior. Therefore, this study is still at an underexplored stage (Um and Zhang, 2021). In order to address this issue, this study analyzes the influencing mechanism of international students’ entrepreneurial behavior from both internal and external aspects.

A review of the existing literature reveals that current research has focused on investigating the driving force of entrepreneurship by determining why individuals have entrepreneurial intention to become entrepreneurs (Kautonen et al., 2015; Fuller et al., 2018). In the research, entrepreneurship models are mainly used to explain the formation of entrepreneurial intention. However, there is very limited attention to the role of entrepreneurial behavior activities (Li et al., 2020) and empirical studies that use entrepreneurial self-efficacy and entrepreneurial intention as serial double mediating variables to study the influencing mechanism of entrepreneurial behavior have gone unnoticed. Therefore, it is crucial to link all these factors to provide new theoretical and practical insights.

Therefore, based on the logical framework of Social Cognitive Theory and Theory of Hierarchy of Needs, this manuscript constructs a mechanism model of entrepreneurial behavior with entrepreneurship education, government support, and global competence as independent variables, entrepreneurial self-efficacy and entrepreneurial intention as mediators, and entrepreneurial behavior of international students in Korea as dependent variable, aimed at providing new theoretical guidance and practical insights for international students in Korea.

## Theoretical Background

### Entrepreneurship Education, Entrepreneurial Self-Efficacy, Entrepreneurial Intention, and Entrepreneurial Behavior

International students in Korea lack resources and experience, therefore it is necessary to provide them with entrepreneurship education, including exploration of entrepreneurial ideas, formulation of specific business plans, and the entrepreneurial knowledge and attitudes required for successful operations.

The Teachability Theory (Drucker, 1985) of entrepreneurship education has laid a theoretical foundation for the academic research on the influencing mechanism of entrepreneurship education on entrepreneurial behavior. Most scholars have given a positive answer to the question whether entrepreneurship can be taught. Entrepreneurship has innate teachability (Hindle, 2007), and the main goal of entrepreneurship education is to influence entrepreneurial behavior (Kolvereid and Isaksen, 2006). Mcmullan’s research also pointed out that a comprehensive approach to education and teaching can meet the different entrepreneurial skills needs of individuals, and then enhance their entrepreneurial spirit and entrepreneurial behavior (Mcmullan and Long, 1987).

Entrepreneurial intention is the “wind vane” of entrepreneurial behavior (Linan, 2008). Ajzen (1991) believed that entrepreneurial intention is a necessary prerequisite for entrepreneurial behavior, and entrepreneurship education has a significant stimulating effect on the generation of entrepreneurial intention. Furthermore, entrepreneurship education can form leading factors for intentions represented by behavioral attitudes, subjective norms and behavioral control, and then have a positive influence on entrepreneurial intention (Christina, 2017). Entrepreneurship education is one of the core factors influencing entrepreneurial intention, and that an individual’s entrepreneurial intention directly determines whether the individual will participate in entrepreneurial behavior (Boahemaah et al., 2020; Ok et al., 2020). Although most scholars have affirmed the teachability of entrepreneurship and pointed out that entrepreneurship education can have a certain impact on individual future entrepreneurial activities, there are currently few studies on the direct relationship between entrepreneurship education and entrepreneurial behavior. And in the results of many previous studies, the relationship of entrepreneurship education and entrepreneurial intention is controversial, so clear identification and confirmation and verification of the positive effects of entrepreneurship education are necessary.

Su and Lim (2020) pointed out that entrepreneurship education is the key to enhancing entrepreneurial self-efficacy and entrepreneurial intention, and also an important means to promote individual participation in entrepreneurship. Entrepreneurial self-efficacy, as a deep-seated belief, can promote the generation of entrepreneurial intention and increase the possibility of individual participation in entrepreneurial behavior (Lee and Chang, 2018). Boyd and Vozikis (1994) indicated that many pre-factors indirectly influence the intensity of individuals’ tendency to participate in entrepreneurial practice through the mediating effect of entrepreneurial self-efficacy. Specifically, an individual’s entrepreneurial self-efficacy can not only influence the formation of its entrepreneurial intention, but also influence the subsequent entrepreneurial behavior, that is, if an individual’s perceived entrepreneurial self-efficacy is high, the individual’s entrepreneurial intention will be relatively strong, and finally the possibility of the individual participating in entrepreneurial practice will be higher. Based on the above research, this study believes that entrepreneurship education can indirectly influence entrepreneurial behavior through entrepreneurial intention, and one of the ways that entrepreneurship education influences entrepreneurial intention is to stimulate individual entrepreneurial intention by increasing individual’s entrepreneurial self-efficacy.

Therefore, the hypotheses are proposed as follows:

• H1: Entrepreneurship education has a significant positive influence on the entrepreneurial behavior of international students in Korea.
• H2: Entrepreneurial intention plays a mediating role in the influence of entrepreneurship education on entrepreneurial behavior of international students in Korea.
• H3: Entrepreneurship education has a significant positive influence on entrepreneurial behavior through the serial double mediating effect of entrepreneurial self-efficacy and entrepreneurial intention.

### Government Support, Entrepreneurial Self-Efficacy, Entrepreneurial Intention, and Entrepreneurial Behavior

Entrepreneurial support is mostly provided by the government. Depending on the perceived usefulness of the entrepreneurial support system and the actual support level, entrepreneurial intention, and even entrepreneurial behavior will be different. The biggest lack of preparation for international students in Korea is that they are unable to accumulate entrepreneurial resources and social connection beforehand in the places where they start their business, and such lack of preparation can seriously influence the entrepreneurial self-efficacy of entrepreneurs. Therefore, there is an urgent need for government support to alleviate the pressure of entrepreneurial start-up and development on entrepreneurs. Government support is mainly reflected in two aspects: firstly, to give policy support, such as non-profit tax reduction and exemption, and to provide partial discount loans; secondly, to provide service support for solving problems, by simplifying the evaluation procedures of entrepreneurial qualifications, broadening the channels of entrepreneurial credit loans, providing entrepreneurial technical guidance and training, and building a business interaction platform conducive to entrepreneurship. Im (2013) found that tax support and financial support have a positive influence on entrepreneurial intention in their study on potential entrepreneurs and mature entrepreneurs.

Does the entrepreneurial self-efficacy of international students in Korea originate from the important characteristics of such students, or can it be achieved through external stimuli? Wang et al. (2020) looked at the entrepreneurial intention of college students and stated that entrepreneurial policy positively and significantly affect the entrepreneurial intention of college students, and entrepreneurial self-efficacy is completely mediated in the relationship between entrepreneurial policy and entrepreneurial behavior. Park et al. (2015) pointed out that educational support, funding support, and marketing support do not directly affect start-up willingness. In the study of Im and Jeon (2015), they analyzed the influence of independent variables on entrepreneurial intention by classifying entrepreneurial support into tax support, financial support, technical support, operation support, and infrastructure support, etc. The results showed that tax support and financial support had a significant influence on entrepreneurial intention, while the influence of technical support, operation support and infrastructure support was not significant. Therefore, direct support such as tax preference or financial preference can strengthen entrepreneurial intention more than indirect support. In addition, Lu et al. (2021) held that entrepreneurship support has a significant strengthening effect on entrepreneurial self-efficacy, and entrepreneurial self-efficacy is completely mediated in the relationship between entrepreneurship support and entrepreneurial behavior.

According to the above research, the hypotheses are proposed as follows:

• H4: Government support has a significant positive influence on the entrepreneurial behavior of international students in Korea.
• H5: Entrepreneurial intention plays a mediating role in the influence of government support on entrepreneurial behavior of international students in Korea.
• H6: Government support has a significant positive influence on entrepreneurial behavior through the serial double mediating effect of entrepreneurial self-efficacy and entrepreneurial intention.

### Global Competence, Entrepreneurial Self-Efficacy, Entrepreneurial Intention, and Entrepreneurial Behavior

The concept of global competence was first put forward by the Council on International Educational Exchange (CIEE) in 1988. Throughout the 1980s and 1990s, American scholars and educators carried out research on global competence, elaborated on its constituent elements, and put forward an indicator system. One of the most influential was the five major components of global competence, namely knowledge, empathy, support, foreign language proficiency, and task performance, first proposed by Richard D. Lambert, former honorary director of the National Foreign Language Center, in 1993. Subsequently, Hunter (2004) detailed the global competence indicator system through empirical research.

Lee and Lee (2016) found a significant positive influence of both knowledgeability and adaptability in global competence on entrepreneurial intention through his study. Throughout the previous studies, there are many studies on the influence of personal and environmental characteristics of future entrepreneurs on their entrepreneurial intention, but there is still insufficient research on the influence of global competence of international students on entrepreneurial intention and behavior. So will the global competence strengthened by international students (as potential entrepreneurs) in the process of studying in Korea influence their future entrepreneurial activities?

Therefore, the hypotheses are proposed as follows:

• H7: Global competence has a significant positive influence on the entrepreneurial behavior of international students in Korea.
• H8: Entrepreneurial intention plays a mediating role in the influence of global competence on the entrepreneurial behavior of international students in Korea.
• H9: Global competence has a significant positive influence on entrepreneurial behavior through the serial double mediating effect of entrepreneurial self-efficacy and entrepreneurial intention.

**FIGURE 1:**
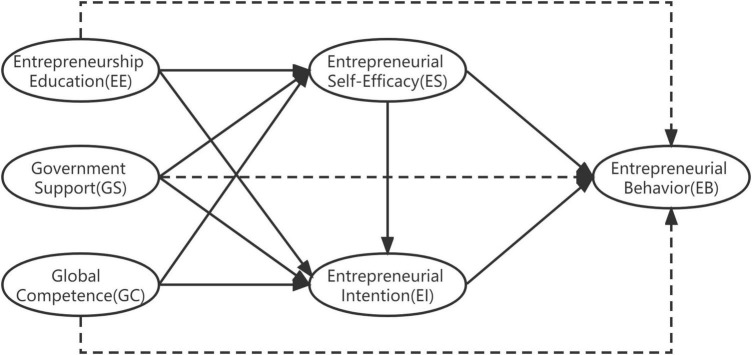
Hypothesized model. Arrows represent hypothesized paths.

In conclusion, this article analyzes the influence of entrepreneurship education, government support and global competence on entrepreneurial behavior, and verify the serial double mediating effect of the self-efficacy and entrepreneurial intention, and its research framework is shown in Figure 1.

## Materials and Methods

### Data Collection and Sample

In this study, international students from the top 10 Korean universities with the highest percentage of international students were selected for the questionnaire survey, using non-probability sampling technique (convenience sampling). In entrepreneurship education studies, convenience sampling is prevalent (e.g., Nowiński et al., 2019). Although non-probability sampling has generalizability limitations, the method still results in quality data when the response rates and participation levels of samples are high (Coviello and Jones, 2004). In order to ensure that the items can be accurately understood by the respondents and avoid deviation, the questionable items (e.g., difficult to understand, unsuitable to answer, ambiguous expression, sensitive item, etc.) of the original questionnaire were modified after small-scale pre-testing. The preliminary questionnaire includes 68 items and is scored on a 5-point scale, from “strongly disagree/know nothing about it” to “strongly agree/know much about it.”

A total of 851 questionnaires from international students in Korea were collected through online survey by use of random sampling, and finally 800 valid questionnaires were obtained by deleting those questionnaires that were not answered completely and carefully, with an effective response rate of 94%. In terms of demographic characteristics, there were slightly more males than females in the survey samples (including 496 males and 304 females); the age of the samples (80%) ranged from 19 to 30 years old. Among them, there were 642 undergraduate students (80.25%) and 158 graduate students (19.75%); in terms of major, there were 123 majoring in humanities, 303 in economics and management, 226 in engineering, 126 in natural science and 22 in others; 491 people’s parents have entrepreneurial behavior, accounting for 61.4%.

### Measures

#### Entrepreneurship Education

The measurement of entrepreneurship education (EE) was mainly based on the relevant research of Jung (2014). It was specifically composed of five dimensions such as business plan, technology, marketing, fund-related issues and guidance, including a total of 18 items. We have used a 5-point scale ranging from 1 = strongly disagree to 5 = strongly agree.

#### Government Support

Government support (GS) scale was mainly based on the research of Park et al. (2015) scale, which included three dimensions of financial support, consulting support, and marketing support. Questions were constructed on a 5-point Likert scale, with answers ranging from 1 = strongly disagree to 5 = strongly agree.

#### Global Competence

The global competence (GC) scale was mainly based on Hunter’s (2004) study and the scale he developed, which specifically consisted of three dimensions: knowledge, technology, and attitude. A 5-point scale was used ranging from 1 = strongly disagree to 5 = strongly agree.

#### Entrepreneurial Self-Efficacy

The measurement of entrepreneurial self-efficacy (ES) mainly drew on the research of Ahn and Park (2018), and consisted of self-confidence, self-regulation efficacy, and job challenge. All the constructs were measured using a 5-point scale, ranging from 1 = strongly disagree to 5 = strongly agree.

#### Entrepreneurial Intention

The entrepreneurial intention (EI) scale was mainly based on the research of Veciana et al. (2005), and included six items such as “I intend to establish a company within 5 years after graduation.” A 5-point scale was used ranging from 1 = strongly disagree to 5 = strongly agree.

#### Entrepreneurial Behavior

The entrepreneurial behavior (EB) scale was mainly based on the studies of Won (2019), and included seven items such as “I have invested a lot of time to realize my entrepreneurial ideas.” All the items were assessed using a 5-point scale, ranging from 1 = strongly disagree to 5 = strongly agree.

### Data Analysis

For the data collected by the questionnaires, SPSS 23.0 was mainly used for data entry, and SPSS and AMOS (IBM Corp., Armonk, NY, United States; Arbuckle, 2016) were used for related data processing and analysis.

#### Common Method Bias Test

Exploratory factor analysis was performed on the variables involved by use of Harman’s single factor test method (Harman, 1976). The first factor explained less than 50% of the variance in total, indicating that there is no serious common method bias. Confirmatory factor analysis (CFA) was performed again on the variables, and the scale fit indices (X^2^/df = 1.488, RMSEA = 0.025, CFI = 0.985, TLI = 0.983, SRMR = 0.029) showed a good fit of the data and the model. In addition, the variance inflation factors (VIF) for all variables are less than 1 (maximum 0.1729), proving that there is no multicollinearity problem among the independent variables.

#### Reliability and Validity Tests

Through reliability analysis, it is concluded that the CITC values of GC4 in global competence (GC) dimensions are all less than 0.5 and the Cronbach’s α value is increased after deleting the questions. Therefore, deletion is made. In Table 1, the Cronbach’s alpha of the variables (i.e., entrepreneurship education, government support, global competence, entrepreneurial self-efficacy, entrepreneurial intention, and entrepreneurial behavior) in this study are 0.919, 0.863, 0.916, 0.910, 0.895, and 0.889, respectively, which are all greater than 0.8. In addition, their composite reliability (CR) are 0.837, 0.756, 0.815, 0.799, 0.897, and 0.890, respectively, which are all greater than 0.7, indicating good reliability of the scale.

**TABLE 1:** Results of tests of reliability and validity.

Constructs		Loadings	Cronbach’s α	CR	AVE
Entrepreneurship education			0.919	0.837	0.507
Business plan	EEB (4)	0.728			
Technology	EET (4)	0.788			
Marketing	EEM (4)	0.723			
Funding	EEF (3)	0.683			
Mentoring	EER (3)	0.630			
Government support			0.863	0.756	0.508
Financial	GSF (3)	0.702			
Consulting	GSC (3)	0.768			
Marketing	GSM (3)	0.665			
Global competence			0.916	0.815	0.596
Knowledge	GCK (3)	0.737			
Skills	GCS (4)	0.870			
Attitudes	GCA (6)	0.699			
Entrepreneurial self-efficacy			0.910	0.799	0.570
Confidence	ESC (5)	0.737			
Self-control efficacy	ESS (5)	0.870			
Task challenge	EST (4)	0.699			
Entrepreneurial intention			0.895	0.897	0.592
EI1		0.752			
EI2		0.732			
EI3		0.801			
EI4		0.757			
EI5		0.794			
EI6		0.777			
Entrepreneurial behavior			0.889	0.890	0.537
EB1		0.747			
EB2		0.746			
EB3		0.736			
EB4		0.740			
EB5		0.801			
EB6		0.706			
EB7		0.644			

*CR, composition reliability; AVE, average variance extracted.*

The dimensional factor loadings of each variable in this study are all greater than 0.6, the average variance extracted (AVE) are 0.507, 0.508, 0.596, 0.570, 0.592, and 0.537, respectively, which are all greater than 0.5 and have good convergent validity. In Table 2, the AVE square root values of the scale factors are all greater than the correlation coefficients of the factors with other factors, indicating good discrimination validity.

**TABLE 2:** Correlations among the variables.

	EE	GS	GC	ES	EI	EB
EE	(0.712)					
GS	0.287**	(0.713)				
GC	0.262**	0.334**	(0.755)			
ES	0.489**	0.486**	0.398**	(0.769)		
EI	0.373**	0.470**	0.386**	0.466**	(0.733)	
EB	0.447**	0.479**	0.476**	0.521**	0.515**	(0.772)

**At the 0.01 level (two-tailed test), the correlation is significant. EE, entrepreneurship education; GS, government support; GC, global competence; ES, entrepreneurial self-efficacy; EI, entrepreneurial intention; EB, entrepreneurial behavior. The diagonal values (in brackets) are the square root of corresponding AVE, and the value on triangle elements are correlations among the variables.

#### Correlation Analysis

Correlation tests were conducted between the variables, and the results in Table 2 showed that there was a significant correlation between the variables (*P* < 0.01), which initially verified the research hypotheses.

#### Path Analysis

**FIGURE 2:**
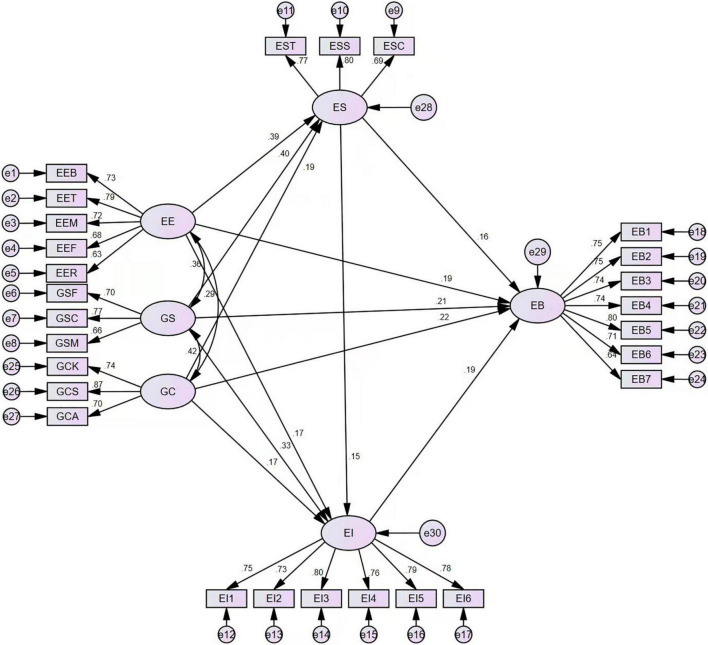
Structural equation modeling (SEM) path analysis.

The data were analyzed through AMOS. The results of structural equation modeling (SEM) are shown in Figure 2.

##### The Direct Influence of Entrepreneurship Education, Government Support, and Global Competence on Entrepreneurial Behavior

Path analysis was used in this study to examine the direct influence of independent variables (entrepreneurship education, government support, and global competence) on entrepreneurial behavior. As shown in Table 3, entrepreneurship education has a significant positive influence on entrepreneurial behavior (β = 0.193, *P* < 0.001), government support is positively and significantly related to entrepreneurial behavior (β = 0.208, *P* < 0.001), and global competence is positively and significantly related to entrepreneurial behavior (β = 0.223, *P* < 0.001). Therefore, hypotheses 1, 4, and 7 are supported.

**TABLE 3:** Results of path coefficient analysis of structural equation model.

Hypotheses	Relationships			Standardization coefficient	S.E.	C.R.	*P*	Result
H1	EE	→	EB	0.193	0.054	4.544	***	Supported
H4	GS	→	EB	0.208	0.069	4.082	***	Supported
H7	GC	→	EB	0.223	0.049	5.731	***	Supported

***P < 0.001.

*EE, entrepreneurship education; GS, government support; GC, global competence; ES, entrepreneurial self-efficacy; EI, entrepreneurial intention; EB, entrepreneurial behavior.*

##### Influence Analysis of Single Mediator

The bias-corrected percentile Bootstrap method in AMOS was used in this study to examine the single mediating effect of entrepreneurial intention in the influence of entrepreneurship education, government support, and global competence on entrepreneurial behavior.

The results of the influence of entrepreneurship education on entrepreneurial behavior with entrepreneurial intention as single mediator are as follows (Table 4): The predictive effect of entrepreneurship education on entrepreneurial intention is significant (β = 0.170, *P* < 0.001), the predictive effect of entrepreneurial intention on entrepreneurial behavior is still significant (β = 0.187, *P* < 0.001), and the direct predictive effect of entrepreneurship education on entrepreneurial behavior is also significant (β = 0.193, *P* < 0.001). The test with Bootstrap method shows that 95% of confidence interval of indirect influence is [0.014, 0.060] (exclusive of 0), while 95% of confidence interval of direct influence is [0.109, 0.273] (exclusive of 0), indicating that there is a partial mediating effect of entrepreneurial intention between entrepreneurship education and entrepreneurial behavior. Therefore, the hypothesis H2 is supported.

**TABLE 4:** Influence test with entrepreneurial intention as single mediator.

	Mediation path	Type	95% CI				Result
			Standardization coefficient	Lower limit	Upper limit	*P*	
Model 1	EE-EB	Direct influence	0.193	0.109	0.273	***	Partial mediating effect; H2 is supported.
	EE-EI-EB	Indirect influence	0.032	0.014	0.060	***	
Model 2	GS-EB	Direct influence	0.208	0.106	0.310	***	Partial mediating effect; H5 is supported.
	GS-EI-EB	Indirect influence	0.063	0.033	0.102	***	
Model 3	GC-EB	Direct influence	0.223	0.146	0.297	***	Partial mediating effect; H8 is supported.
	GC-EI-EB	Indirect influence	0.032	0.016	0.058	***	

***P < 0.001.

*EE, entrepreneurship education; GS, government support; GC, global competence; ES, entrepreneurial self-efficacy; EI, entrepreneurial intention; EB, entrepreneurial behavior.*

The results of the influence of government support on entrepreneurial behavior with entrepreneurial intention as single mediator are as follows: The predictive effect of government support on entrepreneurial intention is significant (β = 0.335, *P* < 0.001), the predictive effect of entrepreneurial intention on entrepreneurial behavior is also significant (β = 0.187, *P* < 0.001), and the direct predictive effect of government support on entrepreneurial behavior is still significant (β = 0.208, *P* < 0.001). The test with Bootstrap method also shows that 95% of confidence interval of indirect influence is [0.033, 0.102] (exclusive of 0), while 95% of confidence interval of direct influence is [0.106, 0.310] (exclusive of 0), indicating that there is a partial mediating effect of entrepreneurial intention between government support and entrepreneurial behavior. Therefore, the hypothesis H5 is supported.

The results of the influence of global competence on entrepreneurial behavior with entrepreneurial intention as single mediator are as follows: The predictive effect of global competence on entrepreneurial intention is significant (β = 0.173, *P* < 0.001), the predictive effect of entrepreneurial intention on entrepreneurial behavior is also significant (β = 0.187, *P* < 0.001), and the predictive effect of global competence on entrepreneurial behavior is still significant (β = 0.223, *P* < 0.001). The test with Bootstrap method shows that 95% of confidence interval of indirect influence is [0.016, 0.058] (exclusive of 0), while 95% of confidence interval of direct influence is [0.146, 0.297] (exclusive of 0), indicating that there is a partial mediating effect of entrepreneurial intention between global competence and entrepreneurial behavior. Therefore, the hypothesis H8 is supported.

##### Influence Analysis of Serial Double Mediator

The bias-corrected percentile Bootstrap method in AMOS was used in this study to examine the double mediating effect of entrepreneurial self-efficacy and entrepreneurial intention, as shown in Table 5.

**TABLE 5:** Influence test with entrepreneurial self-efficacy and entrepreneurial intention as serial double mediator.

	Mediation path	Type	95% CI				Result
			Standardization coefficient	Lower limit	Upper limit	*P*	
Model 4	EE-EB	Direct influence	0.193	0.109	0.273	***	Partial mediating effect; H3 is supported.
	EE-ES-EI-EB	Indirect influence	0.010	0.002	0.023	**	
Model 5	GS-EB	Direct influence	0.208	0.106	0.310	***	Partial mediating effect; H6 is supported.
	GS-ES-EI-EB	Indirect influence	0.010	0.002	0.024	**	
Model 6	GC-EB	Direct influence	0.223	0.146	0.297	***	Partial mediating effect; H9 is supported.
	GC-ES-EI-EB	Indirect influence	0.005	0.001	0.012	**	

**P < 0.01; ***P < 0.001.

*EE, entrepreneurship education; GS, government support; GC, global competence; ES, entrepreneurial self-efficacy; EI, entrepreneurial intention; EB, entrepreneurial behavior.*

Model 4 shows a significant influence of entrepreneurship education exerted on entrepreneurial behavior through entrepreneurial self-efficacy and entrepreneurial intention (β = 0.010, *P* < 0.01), and the direct influence of entrepreneurship education on entrepreneurial behavior is also significant (β = 0.193, *P* < 0.001). The test with Bootstrap method shows that 95% of confidence interval of indirect influence is [0.002, 0.023] (exclusive of 0), while 95% of confidence interval of direct influence is [0.109, 0.273] (exclusive of 0), indicating that there is a partial mediating effect of entrepreneurial self-efficacy and entrepreneurial intention between entrepreneurship education and entrepreneurial behavior. Therefore, the hypothesis H3 is supported.

Model 5 shows a significant influence of government support exerted on entrepreneurial behavior through entrepreneurial self-efficacy and entrepreneurial intention (β = 0.010, *P* < 0.01), and the direct influence of government support on entrepreneurial behavior is also significant (β = 0.208, *P* < 0.001). The test with Bootstrap method shows that 95% of confidence interval of indirect influence is [0.002, 0.024] (exclusive of 0), while 95% of confidence interval of direct influence is [0.106, 0.310] (exclusive of 0), indicating that there is a partial mediating effect of entrepreneurial self-efficacy and entrepreneurial intention between government support and entrepreneurial behavior. Therefore, the hypothesis H6 is supported.

Model 6 shows a significant influence of global competence exerted on entrepreneurial behavior through entrepreneurial self-efficacy and entrepreneurial intention (β = 0.005, *P* < 0.01), and the direct influence of global competence on entrepreneurial behavior is also significant (β = 0.223, *P* < 0.001). It is shown that 95% of confidence interval of indirect influence is [0.001, 0.012] (exclusive of 0), while 95% of confidence interval of direct influence is [0.146, 0.297] (exclusive of 0), indicating that there is a partial mediating effect of entrepreneurial self-efficacy and entrepreneurial intention between global competence and entrepreneurial behavior. Therefore, the hypothesis H9 is supported.

## Conclusion and Discussion

This study analyzes the influence of entrepreneurship education, government support, global competence on entrepreneurial behavior, and reveals the mediating role of entrepreneurial self-efficacy and entrepreneurial intention in this process. The main conclusions include the following.

This study finds that entrepreneurship education, government support, global competence positively affects entrepreneurial behavior, which were accepted. These hypothesizes contribute to the literature of entrepreneurship, because few studies have examined the direct role of entrepreneurship education, government support, global competence on entrepreneurial behavior. The more rational curriculum provision, excellent faculty for entrepreneurship education, perfect system of supporting and resourceful entrepreneurial practice platform, diversified policy support will increase the likelihood of students’ entrepreneurial behavior. Individuals with a high level of global competence are more inclined to accomplish a certain level of task to realize their value. Therefore, our results are consistent with prior research (Rauch and Hulsink, 2015; Cui, 2021; Kim and Lee, 2017).

The research finds that entrepreneurial intention plays an intermediary role in entrepreneurship education, government support, global competence, and entrepreneurial behavior. These findings are similar to previous studies (Ok et al., 2020; Um and Zhang, 2021). This finding is consistent with the theory of planned behavior, where intention predicts the occurrence of actual behavior and behavior is a resultant display of intention. This indicates that support of educational institutions and government departments has a great potential motivational effect on entrepreneurial behavior of students in various aspects. In the early stage of entrepreneurship, students are in lack of space, resources and funds, etc., so government should provide support, such as setting up venture funds to provide certain financial support for entrepreneurial students, or even providing free space to help students find resources (Zhao and Zhao, 2021). This will make students willing to start business and achieve good results in their entrepreneurship. Moreover, when their personal comprehensive qualities and abilities reach the international standards, they can obtain more information and fully understand the current situation and needs of the market, which will help to conceive new business models, strengthen the characteristics and differentiation of the entrepreneurial subjects, enhance their entrepreneurial intentions and promote their entrepreneurial behaviors.

The research also finds that entrepreneurial self-efficacy and entrepreneurial intention exert a partially significant positive serial double mediating effect in the influencing mechanism of entrepreneurship education, government support, and global competence on entrepreneurial behavior. On the one hand, entrepreneurship education and government support directly affects entrepreneurial intention; on the other hand, strong entrepreneurial support enhances the entrepreneurial self-efficacy and entrepreneurial intention of students, so that they can get support and help from the relevant departments when they face difficulties in starting a business, and will turn their entrepreneurial intentions into entrepreneurial behaviors.

In previous studies, scholars used to study the relationship between entrepreneurship education, government support, global competence, and entrepreneurial behavior from a single perspective. However, this study broadens the scope and perspective of previous research, provides an innovative theoretical perspective for a more comprehensive study of the influence of entrepreneurship education on entrepreneurial behavior, and contributes to enriching and improving the research on the factors influencing entrepreneurial behavior.

In addition, although many studies have shown that entrepreneurial intention is a necessary condition and an important influencing factor of entrepreneurial behavior, entrepreneurship education cannot be “dedicated” only to improving entrepreneurial intention or any conception; the breakage or disappearance of any of the influencing factors (antecedent variables) of entrepreneurial behavior will lead to the failure of entrepreneurship education. Education departments and government agencies should be fully aware of this rule, and cultivate and improve the comprehensive quality of students.

## Practical Implications

This study confirms the value of government support, entrepreneurship education and global competence in entrepreneurial activities, and has practical significance to entrepreneurship education, supportive policies, and the development of global competence of students. In this study, the partially positive and significant serial double mediating effect of entrepreneurial self-efficacy and entrepreneurial intention in the influencing mechanism of entrepreneurship education, government support, global competence, and entrepreneurial behavior are highlighted, suggesting that entrepreneurship education, government support, and global competence can significantly influence entrepreneurial behavior indirectly through the serial double mediating effect. Therefore, entrepreneurial self-efficacy is crucial for developing entrepreneurial process to promote entrepreneurial intention and turn it into entrepreneurial behavior, and entrepreneurship education and supportive policies should be enhanced to foster global competence and encourage students to start their own businesses in the future.

First, universities and colleges should adopt different teaching focuses and programs to improve the entrepreneurial self-efficacy of different groups of students through categorized teaching and guidance, so that they can find an effective orientation that meets their entrepreneurial intentions.

Second, universities and colleges should also actively integrate the current entrepreneurship supportive policies and cases into the on-campus entrepreneurship education, so that the support from the external environment of entrepreneurship can be effectively transformed into the improvement of entrepreneurial self-efficacy of college students, which can in turn act positively on their entrepreneurial intentions and behaviors.

Third, it is necessary to make global competence training compulsory in entrepreneurship courses and all education schools, and further integrate the concept of global competence training into the whole process of talent cultivation. In terms of curriculum, global competence is not involved in a single discipline, but requires interdisciplinary cooperation for promotion.

Furthermore, this study is of practical significance to international students. Some scholars believe that study abroad is not only a way for students from different countries to make exchanges, but also an opportunity to develop globally competent human resources for the country and the world. Cross-cultural communication and collaboration emphasized in global competence require individual’s perception and understanding of alien cultures, and study abroad is an effective way to achieve this goal. Therefore, it is natural for students to enhance their global competence to some extent in the process of study abroad, and effectively turn it into an increase in their entrepreneurial self-efficacy, which will in turn act positively on their entrepreneurial intentions and increase the conversion rate of their entrepreneurial behaviors.

## Limitations and Future Directions

First, there are limitations on the study samples. In this study, the respondents are the international students of general universities and colleges, and the students of higher vocational and technical colleges are not involved. Entrepreneurship education should be systematic and coherent, so the students of primary and secondary schools shall also be involved in the sample collection. Students of higher vocational and technical colleges and primary and secondary schools are not the objects of this study; however, research can be conducted on them in the future. More research on entrepreneurship education in higher vocational and technical colleges and primary and secondary schools shall be conducted to lay the groundwork for incorporating entrepreneurship education into the national education system.

Second, the follow-up research is not in place. The sample collection of this study only comes from a period of survey, and the entrepreneurial status and intention of the respondents may change over time. Therefore, in the related research in the future, a sample of relatively fixed entrepreneurial team of international students can be tracked for the follow-up study to investigate their various performance behaviors in social entrepreneurship and demands of entrepreneurship education, and listen to their suggestions on entrepreneurship education in universities and colleges based on their own conditions, so as to continuously clarify the various possible influencing mechanism relationships of entrepreneurship education on entrepreneurial behavior and improve the relevant conclusions.

Finally, as technology advances, the way people teach and learn changes. Entrepreneurship education should incorporate innovative technologies and such as providing more free online courses and services on educational issues (Gunkel, 2017). These innovative technologies can provide greater space for entrepreneurship education in the future.

## Data Availability Statement

The original contributions presented in the study are included in the article/supplementary material, further inquiries can be directed to the corresponding author.

## Author Contributions

JZ and BL designed the study, performed the statistical analysis, formulated the conclusions and wrote the manuscript with the equal contribution. YZ conducted the research and revised the manuscript. CG contributed greatly to the overall planning of the study and edited the manuscript. ZL conceived and designed the study, participated in its coordination, and edited the manuscript. All authors read and approved the final manuscript.

## Conflict of Interest

The authors declare that the research was conducted in the absence of any commercial or financial relationships that could be construed as a potential conflict of interest.

## Publisher’s Note

All claims expressed in this article are solely those of the authors and do not necessarily represent those of their affiliated organizations, or those of the publisher, the editors and the reviewers. Any product that may be evaluated in this article, or claim that may be made by its manufacturer, is not guaranteed or endorsed by the publisher.
